# Miscellaneous Notes

**Published:** 1875-05

**Authors:** 


Dr. John T. Dixon, of Meriwether county, Georgia, writes us that he has picked up several papers and a photograph, which were blown by the late tornado from the wrecked house of a friend in Harris county, who, most unfortunately, lost his wife and three children in the fearful war of the elements. It is not a little singular that the articles found by Dr. Dixon were but slightly soiled in their furious transit of thirty miles.

We Learn that the wife of Mr. W. T. Chappell, near Rocky Mount, in Meriwether county, brought forth two girls and a boy on the 14th inst. The mother and offspring are doing well. Dr. J. J. Addy, Drs. J. P. and J. W. Taylor and Dr. W. H. Hardison were the physicians in attendance.

An Offer.—To the first one of our old subscribers who will send us a single copy of the number of this Journal for October, 1861, we will credit upon our books *an entire year's subscription.* Who will send it?

